# Exosome Production Is Key to Neuronal Endosomal Pathway Integrity in Neurodegenerative Diseases

**DOI:** 10.3389/fnins.2019.01347

**Published:** 2019-12-12

**Authors:** Paul M. Mathews, Efrat Levy

**Affiliations:** ^1^Center for Dementia Research, The Nathan S. Kline Institute for Psychiatric Research, Orangeburg, NY, United States; ^2^Department of Psychiatry, New York University Langone Health, New York, NY, United States; ^3^NYU Neuroscience Institute, New York University Langone Health, New York, NY, United States; ^4^Department of Biochemistry and Molecular Pharmacology, New York University Langone Health, New York, NY, United States

**Keywords:** endosome, lysosome, extracellular vesicle, multi-vesicular body, neurodegeneration, apolipoprotein E, Alzheimer’s disease, Down syndrome

## Abstract

Dysfunction of the endosomal–lysosomal system is a prominent pathogenic factor in Alzheimer’s disease (AD) and other neurodevelopmental and neurodegenerative disorders. We and others have extensively characterized the neuronal endosomal pathway pathology that results from either triplication of the amyloid-β precursor protein (APP) gene in Down syndrome (DS) or from expression of the apolipoprotein E ε4 allele (APOE4), the greatest genetic risk factor for late-onset AD. More recently brain exosomes, extracellular vesicles that are generated within and released from endosomal compartments, have been shown to be altered in DS and by APOE4 expression. In this review, we discuss the emerging data arguing for an interdependence between exosome production and endosomal pathway integrity in the brain. *In vitro* and *in vivo* studies indicate that altered trafficking through the endosomal pathway or compromised cargo turnover within lysosomes can affect the production, secretion, and content of exosomes. Conversely, exosome biogenesis can affect the endosomal–lysosomal system. Indeed, we propose that efficient exosome release helps to modulate flux through the neuronal endosomal pathway by decompressing potential “traffic jams.” Exosome secretion may have the added benefit of unburdening the neuron’s lysosomal system by delivering endosomal–lysosomal material into the extracellular space, where other cell types may contribute to the degradation of neuronal debris. Thus, maintaining robust neuronal exosome production may prevent or mitigate endosomal and lysosomal abnormalities linked to aging and neurodegenerative diseases. While the current evidence suggests that the exosomal system in the brain can be modulated both by membrane lipid composition and the expression of key proteins that contribute to the formation and secretion of exosomes, how exosomal pathway-regulatory elements sense and respond to perturbations in the endosomal pathway is not well understood. Based upon findings from the extensively studied DS and APOE4 models, we propose that enhanced neuronal exosome secretion can be a protective response, reducing pathological disruption of the endosomal–lysosomal system in disease-vulnerable neurons. Developing therapeutic approaches that help to maintain or enhance neuronal exosome biogenesis and release may be beneficial in a range of disorders of the central nervous system.

## Introduction

In Alzheimer’s disease (AD), early alterations of the endosomal system in neurons is followed by extensive disruption of autophagic and lysosomal compartments (Colacurcio et al., 2018). Neuronal endosomal pathway disruption has been suggested to result from multiple pathological insults in AD, including, for example, intravesicular amyloid-β (Aβ) (Willen et al., 2017). Our laboratories and collaborators have focused on the apparently Aβ-independent endosomal pathway changes that are seen in Down syndrome (DS) (Cataldo et al., 2000, 2003, 2008) and as a result of the expression of the apolipoprotein E ε4 allele (ApoE4) (Nuriel et al., 2017). In DS, which leads to early onset AD, the elevated levels of the β-site cleaved carboxyl-terminal fragment (βCTF) of the amyloid-β precursor protein (APP) that result from the triplication and overexpression of the APP gene are sufficient to cause early endosomal changes (Cataldo et al., 2003; Salehi et al., 2006; Jiang et al., 2010, 2016; Nixon, 2017). Expression of APOE4, the most important genetic risk factor for late-onset AD (Corder et al., 1993; Farrer et al., 1997; Bu, 2009; Liu et al., 2013), leads to morphologically similar neuronal endosomal pathway changes in humans as well as in mouse models (Cataldo et al., 2000; Nuriel et al., 2017; Zhao et al., 2017). More recently, research focus in these models of AD-related endosomal disruption has extended to exosomes, membrane-bound vesicles that are generated within the endosomal pathway and secreted into the extracellular space (Kreimer et al., 2015; van der Pol et al., 2015). A widely held opinion is that extracellular vesicles (EVs), including exosomes, can be harmful within the brain. These stable vesicles may allow toxic material to be transported between cells and brain regions, and may promote the accumulation of this material in the extracellular space (Valdinocci et al., 2017; Perez et al., 2019).

Nevertheless they may also be beneficial, discarding potentially toxic material that a neuron has targeted for degradation, as well as through the transport of neuroprotective proteins. Indeed, exosome secretion was originally described as a process that can complement and supplement lysosomal and proteasomal degradation for the removal of obsolete membrane and cytosolic materials (Johnstone et al., 1987). While the *in vivo* functions of exosomes in the brain are likely to be a mixture of beneficial and potentially pathological effects, in this review, we have chosen to emphasize the beneficial role that exosome production may play in supporting neuronal endosomal–lysosomal function. Emerging evidence now links endosomal pathway function and the generation and secretion of exosomes into the brain extracellular space. Perturbations of the neuronal endosomal–lysosomal pathway, which can alter endosomal pathway flux and lead to inefficient degradation in lysosomes, appear to affect exosome secretion. Additionally, it appears that disease-driven deficiencies in exosomal production can negatively affect flux and catabolism through the endosomal–lysosomal pathway. Thus, our hypothesis is that exosome production plays a key role in maintaining neuronal endosomal pathway integrity and that disruption of these integrated systems can contribute to neurodegenerative diseases.

## Discussion

### Extracellular Vesicles in the Brain

**FIGURE 1:**
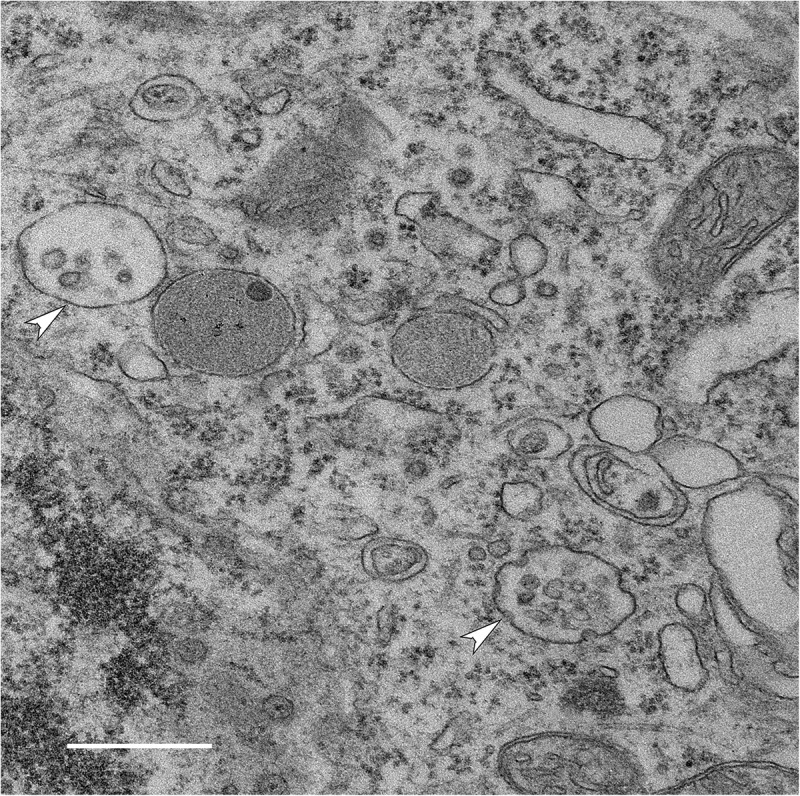
Generation of intraluminal vesicles (ILVs) within MVBs in a neuron. Electron micrograph of MVBs (arrowheads) in a neuron in the brain of a wild-type mouse (bar 500 nm) shows multiple ILVs along with invaginations of the MVB limiting membrane that are likely to represent the formation of nascent ILVs. Once released into the extracellular space, these ILVs are exosomes.

Extracellular vesicles are secreted into tissue extracellular space, biological fluids, and, in culture, conditioned media. Their membrane is rich in phospholipids and they contain lipids, proteins, and RNA (mRNA and miRNA). Multiple types of EVs have been described with different sites of cellular origin (reviewed in van der Pol et al., 2012; Kowal et al., 2014; Kalra et al., 2016) and with distinct molecular and biological properties (Lai et al., 2016; Willms et al., 2016). *Microvesicles* derive from the plasma membrane, have a diameter of 100–1000 nm, and are continuously released from the cell membrane of apparently all cells, although under pathological conditions their release from the cell can also be triggered (Borroto-Escuela et al., 2015). The *exosome* is the most extensively studied EV species, 20–150 nm vesicles formed by the intraluminal invagination of the limiting-membrane of the late endosome/multi-vesicular body (MVB; so named because of the presence of these nascent vesicles within the larger endosomal lumen) (reviewed in Kreimer et al., 2015; van der Pol et al., 2015; Figure 1). Exosomes are these intraluminal vesicles (ILVs) once they are released into the extracellular space upon fusion of MVBs with the plasma membrane (Colombo et al., 2014). During the budding of both microvesicles and ILVs cytosolic content is captured within the lumen of the inchoate vesicle, contributing to the vesicle’s eventual content. Additionally, these vesicles contain membrane lipids and various membrane-associated molecules, some of which are unique to each vesicle subtype. In addition to microvesicles and exosomes, lysosomal exocytosis releases lysosomal luminal contents into the extracellular space. This is, in part, a calcium-regulated process that entails fusion of the lysosomal limiting membrane with the plasma membrane (Stinchcombe et al., 2004). Lysosomes can also transiently contain ILVs delivered from the MVB which are released into the extracellular space upon lysosomal exocytosis (Migliano and Teis, 2018). Dysfunction of the endosomal–lysosomal pathway in AD appears to cause lysosomal components to leak into the extracellular fluid around amyloid plaques and into the cerebrospinal fluid (CSF) (Schwagerl et al., 1995; Nixon and Cataldo, 2006; Colacurcio et al., 2018). Lysosomal proteins, such as cathepsins B and D and lysosome-associated membrane protein 1 (LAMP-1) were identified in brain EVs, suggesting that autophagic-lysosomal dysfunction of neurons in AD may result in vesicular lysosomal exocytosis (Andrews, 2000; Ihara et al., 2012; Urbanelli et al., 2013). Additionally, higher levels of autophagic- and lysosomal-related proteins have been found in neuronal exosomes isolated from the blood in patients with AD compared to non-demented control individuals (Goetzl et al., 2015).

Microvesicles, exosomes, and vesicles resulting from lysosome secretion are typically co-isolated by experimenters from tissues, biological fluids such as plasma, CSF and urine, and from cell-culture conditioned media. These complex EVs preparations can be further purified to enrich for exosomes (Pérez-González et al., 2012, 2017). Exosomes themselves can be specifically identified by various markers such as the transmembrane tetraspanin proteins (e.g., CD63) and the endosomal sorting complex required for transport (ESCRT) machinery proteins (e.g., tumor susceptibility gene 101, TSG101 and ALG-2-interacting protein X, Alix, also called programmed cell death 6-interacting protein, PDCD6IP) involved in ILV formation (van Niel et al., 2018). Nevertheless, a full characterization and cataloging of the various types of EVs found in the brain has not been completed, and any experimental preparation at this point should be assumed to contain more than one type of EVs. An additional complexity is the multiple cell types within the brain that are potential sources of EVs. In this discussion, we have generally defaulted to the inclusive terminology of EVs except when the experimental evidence specifically supports changes in exosomes: we present such data as exosome findings.

### Potential Contribution of EVs to the Spread of Pathogenic Molecules Within the Brain

Extracellular vesicles appear to have roles in cell signaling functions (Record et al., 2011), by shuttling cargo between cells and between tissues (Smalheiser, 2007). In the brain, this may contribute to the regulation of neurotransmitter receptor levels at the synapse (Faure et al., 2006), synapse number (Lee et al., 2018), the production and turnover of myelin membrane proteins (Bakhti et al., 2011; Kramer-Albers and Hill, 2016; Tassew et al., 2017), as well as, unfortunately, the progression and propagation of neurodegenerative diseases (Gould et al., 2003; Vella et al., 2008; Izquierdo-Useros et al., 2011; Budnik et al., 2016). The potential for deleterious functions of EV movement between cells was initially based upon the idea that EVs can efficiently transfer pathogens between cells. In the brain, one such pathogenic molecule that uses EV release is misfolded prion protein (PrP), causing transmissible neurodegenerative diseases such as Creutzfeldt–Jakob disease in humans and bovine spongiform encephalopathy in cattle (Vella et al., 2008; Vilette et al., 2018). α-synuclein, involved in the pathogenesis of Parkinson’s disease, is also secreted in a calcium-dependent manner via EVs (Emmanouilidou et al., 2010; Alvarez-Erviti et al., 2011; Danzer et al., 2012; Stuendl et al., 2016; Ngolab et al., 2017), and the transfer of α-synuclein fibrils between cells appears to be an important component of α-synuclein pathology, spread of the misfolded protein to distant sites, and the activation of immune cells (reviewed in Tofaris, 2017; Grozdanov and Danzer, 2018; Stefanis et al., 2019). In Parkinson’s disease, mutations in the leucine-rich repeat kinase 2 (LRRK2) gene are a frequent genetic cause of the disorder, and it has also been suggested that EV-associated LRRK2 carries pathogenic-potential for the disease (reviewed in Wang and West, 2019).

In AD, EVs have been proposed to contribute to the spread of Aβ and Aβ pathology in the brain (Rajendran et al., 2006) as well as the spread of hyper-phosphorylated pathogenic tau between neurons (Saman et al., 2012; Wang et al., 2017; Guix et al., 2018; Winston et al., 2019). We and others have shown that EVs contain full-length APP, the CTFs of APP generated through cleavage by β-secretase (βCTFs) or α-secretase (αCTFs), Aβ, and the enzymes that are responsible for these cleavage steps (Rajendran et al., 2006; Vingtdeux et al., 2007; Escrevente et al., 2008; Sharples et al., 2008; Pérez-González et al., 2012; Laulagnier et al., 2018; Miranda et al., 2018). The endosomal pathway is critical for the processing of APP (Nixon, 2017), with β-secretase cleavage mediated by BACE1 occurring in endosomes (Koo, 2002; Kinoshita et al., 2003; Small and Gandy, 2006). Thus, the finding that endosome pathway-derived exosomes contain abundant CTFs is not unexpected (Pérez-González et al., 2012; Gauthier et al., 2017). In addition to being the precursor of the neurotoxic Aβ, the βCTF itself has the potential to be a neurotoxic protein that causes neuronal endosomal–lysosomal abnormalities (Jiang et al., 2010, 2016, 2019; Nixon, 2017) and functional sequelae including memory loss (Neve et al., 1996; Oster-Granite et al., 1996; Neve and Robakis, 1998; McPhie et al., 2001; Jiang et al., 2010; Deyts et al., 2012; Flammang et al., 2012; Lauritzen et al., 2012, 2016; Oules et al., 2012; Tamayev and D’Adamio, 2012; Tamayev et al., 2012). The propagation of βCTFs via exosomes from neuron to neuron may have deleterious effects separate from the propagation of Aβ by these vesicles.

### EV Production Within the Brain May Also Be Protective

While it has been shown that EVs can propagate Aβ within the brain (Howitt and Hill, 2016; Xiao et al., 2017), a study has shown that exogenous EVs can paradoxically clear Aβ from the brain of APP overexpressing transgenic mice (Yuyama et al., 2014). *In vitro*, EVs released by neuroblastoma and primary cortical neurons can bind Aβ and can support Aβ fibril formation on their surface (Yuyama et al., 2012). While consistent with the proposed Aβ seeding properties of EVs, EV-bound Aβ can also be taken up by microglia for degradation. *In vivo*, continuous intracerebral infusion of exogenous neuroblastoma-produced EVs apparently trapped and targeted for microglial phagocytosis sufficient Aβ in the brain of APP overexpressing transgenic mice to reduce Aβ levels, amyloid deposition, and synaptotoxicity in the hippocampus (Yuyama et al., 2014). These findings support the idea that the balance that determines whether EVs drive pathology spread or serve as a pathway for the removal of toxic molecules within the brain is likely to be dependent on how efficiently EVs are removed from the brain parenchyma.

The release of the materials contained in an EV may be of benefit to the cell releasing this material. We advance the idea that the appropriate modulation of exosome production is essential to the normal functioning of the endosomal–lysosomal pathway in a neuron. We argue for three overlapping mechanisms by which exosome production can be supportive of the function of the neuronal endosomal–lysosomal system: (1) the transport of beneficial endosomal–lysosomal cargo within the brain; (2) the removal from the lysosomal system of a neuron of potentially deleterious material for degradation by other cells within the brain; and (3) the maintenance of membrane and vesicular-content flux through a neuron’s endosomal–lysosomal pathway.

#### Transporting Protective Endosomal–Lysosomal Cargo Within the Brain

Exosomes are unique in that their limiting membrane renders them more stable in the extracellular environment than is the case for secreted, soluble proteins (reviewed in Simpson et al., 2008). Therefore, the association of neuroprotective/neurotrophic proteins with exosomes likely prolongs the extracellular survival of these proteins, extending their ability to exert protective effects both over time and spatially within the brain. An illustrative example is the cysteine-protease inhibitor cystatin C (CysC), which is contained within the endosomal–lysosomal pathway and implicated in neuroprotection and repair in the nervous system in response to diverse insults (reviewed in Tizon and Levy, 2006; Gauthier et al., 2011; Mathews and Levy, 2016). CysC is also secreted from cells, both in a soluble form and in association with EVs, including exosomes (Ghidoni et al., 2011). We have recently demonstrated that CysC-containing, exosome-enriched EVs protect cultured cells from nutrient deprivation-induced death (Pérez-González et al., 2019). While both CysC-containing and CysC-deficient EVs were taken up by the cultured cells, only EVs containing CysC protected the cells. Interestingly, both exogenous CysC and transgene-mediated overexpression of CysC can promote the secretion of EVs by cultured cells (Pérez-González et al., 2019). Transgene overexpression of CysC also leads to higher EV levels in the brain, showing that this effect of CysC expression occurs *in vivo* (Pérez-González et al., 2019). Thus, the release of EV-associated CysC may lead to a positive-feedback loop further amplifying the protective effects of CysC within the brain. The exosome association of CysC and other neurotrophic proteins such progranulin, which is also both secreted and localized to the endosomal pathway (Benussi et al., 2016), likely prolongs the extracellular survival of these proteins, extending their ability to exert beneficial effects. We have shown that EVs secreted by CysC-deficient cells, subsequently loaded with exogenous CysC, significantly protected primary neurons from nutrients-deprivation-induced death (Pérez-González et al., 2019), revealing the potential for the loading of EVs with CysC and other neuroprotective proteins for clinical purposes.

#### Removal of Endosomal–Lysosomal Material From Neurons for Uptake by Phagocytic Cells and Subsequent Degradation

The production of exosomes moves lipids and proteins from the endosomal–lysosomal pathway into the extracellular space. Exosomes released by a neuron that are degraded by non-neuronal cells such as microglia or otherwise removed from the brain would result in the net elimination of neuronal endosomal–lysosomal content (Yuyama et al., 2012; Joshi et al., 2015). This would also limit neuron-to-neuron propagation of pathological molecules via exosomes (Yuyama et al., 2012; Joshi et al., 2015). Some studies have shown that EV uptake by microglia is more efficient than that by neurons (Fitzner et al., 2011; Yuyama et al., 2012). However, the relative contribution of different cell types to brain exosome uptake is not clear, and neuron-to-neuron exosome trafficking has been shown (Chivet et al., 2014). Indeed, the balance of beneficial clearance versus pathogenic propagation mediated by exosomes and EVs more generally is likely to be complex and dynamic over time, dependent upon such factors as outward CSF and interstitial fluid flux, localized inflammation and gliosis, and the activation status of microglia. As better tools are developed for labeling and tracking EVs *in vivo*, more details will emerge regarding the metabolism of brain EVs and the impact of aging and neurodegenerative diseases upon these processes.

#### Maintaining Flux Through the Endosomal-Autophagy-Lysosomal System

As previously noted, a fundamental effect of exosome production is to remove lipids and proteins from a cell’s endosomal–lysosomal pathway. Our recent findings, particularly in DS and APOE4 carriers, support the idea that exosome release plays an important role in maintaining endosomal–lysosomal function by allowing for an “outlet” other than degradation within the lysosome. We expand upon these findings in support of this idea throughout the following discussion, first summarizing the endosomal-autophagic-lysosomal changes seen in AD and then the effect DS and APOE4 expression has on exosome biology and the impact this has on the endosomal–lysosomal pathway.

### AD Risk, Abnormalities of the Neuronal Endosomal–Lysosomal and Exosome Generation and Release

The neuronal endosomal–lysosomal and autophagy systems are vulnerable to pathogenic changes in AD (Troncoso et al., 1998; Cataldo et al., 2000, 2004; Nixon et al., 2008). Alterations in early and late endosomes include morphologic changes such as enlargement and increased endosome numbers (Cataldo et al., 2000), and are likely to include alterations in endocytosis and flux through endosomal compartments (Cataldo et al., 2008; Small, 2008; Jiang et al., 2010). Autophagic vacuoles accumulate in neurons during AD, most prominently in neuronal processes such as axons, often in proximity to β-amyloid plaques (Yang et al., 2011). Lysosomal alterations were the first AD-driven changes in this system to be described, and include proliferation of lysosomes and increased levels of lysosomal proteins, including degradative lysosomal hydrolases (Cataldo et al., 1995). Findings in mouse models as well as the progression of these changes as seen in human AD are generally consistent with an underlying failure of efficient lysosomal hydrolysis and of autophagosome-lysosome fusion. This failure in turnover is thus an important contributor to both autophagosome accumulation and the proliferation of apparently degradation-deficient lysosomes loaded with undigested material (Colacurcio et al., 2018). AD appears to compromise the efficient movement of cargo through the lysosomal system and its subsequent degradation.

Neuronal endosome enlargement and proliferation appears to be an earlier event than either autophagy or lysosome dysfunction, and endosomal pathway alterations are uniformly seen in late-onset AD cases (Cataldo et al., 2000; Colacurcio et al., 2018). While multiple mechanisms are likely to contribute to the vulnerability of this pathway in neurons during aging and early in AD pathobiology, two extensively studied genetic causes of AD-like endosomal changes are chromosome 21 trisomy in DS (Cataldo et al., 2000, 2004, 2008; Takahashi et al., 2004; Jiang et al., 2010) and inheritance of the APOE4 allele (Cataldo et al., 2000; Nuriel et al., 2017; Zhao et al., 2017), the most important genetic risk factor for AD (Corder et al., 1993; Farrer et al., 1997; Bu, 2009; Liu et al., 2013).

Once early endosomal cargoes are delivered to the late endosome there are two possible fates for these proteins and lipids: either lysosome degradation or packaging into exosomes for release. Exosome secretion has the potential to allow for flux through the endosomal–lysosomal pathway that is independent of lysosomal delivery. Moreover, a neuron’s secreted exosomes can be degraded by other cells or removed from the brain into the periphery. Since the late endosome/MVB regulates endosomal trafficking to the lysosome as well as exosome production, it is not unexpected that changes in brain exosomes have been seen in systems showing AD-like endosomal–lysosomal pathway alterations. Our studies have focused on the effects of DS and APOE4 genotype upon brain exosomes, where we have identified changes in brain exosome production and secretion as well as changes in their protein and lipid constituents (Gauthier et al., 2017; Peng et al., 2019).

A similar integrated relationship has been well established between autophagy and the exosome pathway. Exosome biogenesis and autophagy are linked by their function: exosomes remove cytoplasmic cargo into the extracellular space while autophagy removes cytoplasmic components via lysosome-dependent degradation. Recent studies have revealed shared molecular machinery between exosome biogenesis and autophagy, as well as substantial crosstalk between these two processes via fusion of autophagosomes with MVBs to form amphisomes (reviewed in Xu et al., 2018). For example, knockout of the ESCRT member Alix, which is important for ILV formation, reduces not only exosome generation but also basal autophagy flux, demonstrating a shared regulation between autophagy and exosome biogenesis (Murrow et al., 2015).

Additionally, exosome release is negatively regulated by the mechanistic target of rapamycin complex 1 (mTORC1), the protein complex containing mTOR that acts as the cell’s master nutrient and energy sensor to regulate protein synthesis and autophagy. Sustained activation of mTORC1 reduces the release of exosomes by cells and in animal models, resulting in the intracellular accumulation of ILVs. Conversely, exosome release is stimulated by inhibition of mTORC1 by rapamycin or nutrient and growth factor deprivation (Zou et al., 2019). Inhibition of autophagy by wortmannin or CRISPR/Cas9-mediated knockout of the autophagy protein Atg5 (autophagy-related 5) in neuronal cell lines increased the release of exosomes, documenting this interrelationship in neurons as well as other cell types (Dias et al., 2016).

A pathology relevant interrelationship between exosomal release and autophagy has been seen in cell culture models of α-synuclein aggregation and in α-synuclein transgenic mice: inhibiting autophagy with bafilomycin A reduced the intracellular aggregation of α-synuclein but increased the secretion of smaller oligomers that were predominantly released through exosomes (Poehler et al., 2014). Poehler et al. (2014) suggested that impaired autophagy in the diseased brain not only limits intracellular degradation of misfolded proteins such as α-synuclein, but can also lead to detrimental effects in the local microenvironment due to enhanced α-synuclein secretion. Similarly, inhibition of autophagosome formation by silencing ATG5 in α-synuclein, overexpressing neurons increased the secretion of exosome-associated α-synuclein while blocking exosomal secretion using GW4869 exacerbated α-synuclein-induced cell death (Fussi et al., 2018). Thus, exosomal secretion of α-synuclein is apparently increased after impaired formation of autophagosomes, which can reduce intracellular α-synuclein burden and limit α-synuclein-induced neuronal cell death (Fussi et al., 2018). These findings document the interdependence of autophagy and the exosomal pathway while also highlighting the sometimes contradictory outcome of exosome release. Under pathological conditions, dysfunction of the endosomal–lysosomal and/or autophagic pathways can lead to changes in exosome generation and secretion, which may help to preserve needed flux through the system as a whole. This enhanced exosome production may be protective for the cell secreting the exosomes, yet potentially damaging to neighboring cells and within the local microenvironment.

### Exosome Secretion Is Enhanced in the DS Brain

**FIGURE 2:**
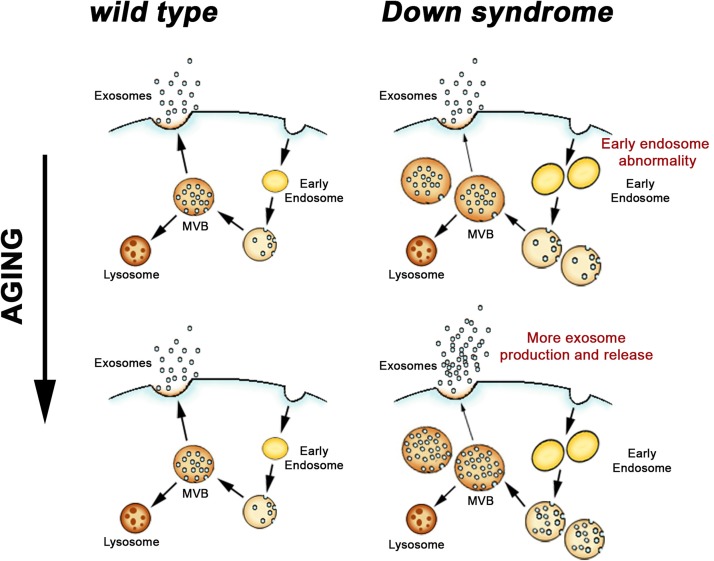
Endosomal and exosomal changes in the DS brain. Endocytosed material is sorted through early endosomes and late endosomes/MVB for either degradation in lysosomes or exosomal secretion. In DS, where neuronal endosome dysfunction begins before birth, an age-dependent increase in exosome release appears to be a compensatory response to limit the extent of the endosomal pathway pathology that results from DS genetic effects.

Given that an increase in early endosomal drive was demonstrated in DS, one would predict a necessary compensatory increase in either lysosome degradation or exosome release. Indeed, enhanced exosome secretion in DS would help to shed excessive neuronal endosomal content into the brain extracellular space (Figure 2). Endosomal pathology has been shown in human DS neurons, in neurons of DS mouse models, and in human DS fibroblasts in culture (Cataldo et al., 2000, 2004, 2008; Takahashi et al., 2004; Jiang et al., 2010). We have shown that higher levels of exosomes occur in each of these systems. In samples of frontal cortices of DS patients who did not have β-amyloid pathology, higher exosome levels in the brain parenchyma were found compared to age-matched diploid controls (Gauthier et al., 2017). Higher exosome levels were also found in the brain extracellular space of two DS mouse models, Ts[Rb(12.17^16^)]2Cje (Ts2) and Ts65n, as compared to diploid littermate controls (Gauthier et al., 2017). Examination of exosome secretion into the media of cultured human fibroblasts showed that these DS fibroblasts, which have AD-like endosomal abnormalities (Cataldo et al., 2008), secrete more EVs compared to age-matched diploid control fibroblasts (Gauthier et al., 2017).

While higher levels of exosomes in the growth media of DS fibroblast is consistent with the idea that DS cells produce greater numbers of exosomes, levels of exosomes in the brain are dependent upon both production and turnover. Recent findings argue that it is brain exosome production, and more specifically neuronal exosome production, which is higher in DS compared to diploid controls. Using high-resolution electron microscopy and quantitative analyses of MVBs in neurons of the frontal cortex of Ts2 mice, we recently showed that Ts2 MVBs are larger, more abundant, and contain a higher number of ILVs per neuron compared to littermate diploid controls (D’Acunzo et al., 2019). While not neuron specific, higher levels in Ts2 mice compared to controls of ILV proteins within the brain subcellular fractions that also contained endosomal markers corroborated these electron microscopy findings (D’Acunzo et al., 2019).

Further evidence for higher exosome production in DS has been provided by examining the expression of key molecular regulators of exosome biogenesis. The formation of ILVs in the late endosome/MVB is regulated by the ESCRT machinery and by an ESCRT independent system that includes tetraspanins (van Niel et al., 2011). Unlike most tetraspanins that are present in the plasma membrane, CD63 is uniquely enriched in the membrane of MVBs and of secreted exosomes (Pols and Klumperman, 2009; van Niel et al., 2011). Several Rab GTPases and SNARE proteins regulate the intracellular trafficking of MVBs toward the plasma membrane for fusion (Eitan et al., 2016). Among them, rab35 likely plays a role in the docking of MVBs to the plasma membrane (Hsu et al., 2010; Klinkert and Echard, 2016). Given the important role that these proteins play in exosome generation and secretion, changes in their expression levels within a cell or tissue are likely to reflect changes in ILV biogenesis and/or exosome secretion. While the expression levels of the ESCRT machinery proteins Alix and TSG101 did not differ in the brains of human DS patients and in DS fibroblasts as compared with diploid controls, the expression of other regulators of exosome production did differ. We have shown higher levels of CD63 and rab35 proteins in brain-tissue homogenates from human DS patients and the Ts2 mouse model compared to diploid controls (Gauthier et al., 2017). CD63 mRNA is up-regulated in the hippocampus of Ts2 mice (Gauthier et al., 2017), arguing that higher levels of CD63 in the brain represents higher expression levels of tetraspanin machinery driving ILV formation, not simply higher amount of CD63 associated with brain exosomes. In contrast, rab35 mRNA is not higher in the Ts2 hippocampus as compared with controls. Unlike CD63, rab35 is not tethered to secreted exosomes, remaining within the cell’s cytoplasm after the fusion of the late endosome/MVB with the plasma membrane and therefore is found in secreted exosomes only in modest amounts (Gauthier et al., 2017). Thus while the amount of rab35 protein is higher, the cell’s need for an ongoing, robust increase in rab35 expression is likely to be less than the need for CD63, which is consumed during exosome production, a difference that is reflected in the greater change in CD63 mRNA levels.

Together these findings in the brain support our model that exosome production itself is higher in DS (Figure 2) and are consistent with the finding that DS fibroblasts secret more exosomes into the cultured media compared to diploid control cells. Like DS brain, CD63 is overexpressed in DS fibroblasts (Gauthier et al., 2017). Importantly, while knockdown of CD63 expression in fibroblasts using interference RNA (siRNA) did not affect exosome secretion by wild-type diploid cells, reducing CD63 expression in DS cells led to reduced exosomes secretion into the cell culture media (Gauthier et al., 2017).

In addition to confirming that higher CD63 expression is necessary for the higher levels of exosome production by DS cells, this manipulation showcased the interdependence between endosomal pathway function and exosome production and secretion. While CD63 knockdown had no effect on exosome production or endosome morphology in diploid cells, CD63 knockdown led to a worsening of the early endosomal phenotype in DS fibroblasts, including further enlargement and increased numbers (Gauthier et al., 2017). This argues that partially blocking exosome generation in DS cells increases the intracellular accumulation of endosomal compartments, worsening the DS endosomal pathology. In contrast, silencing CD63 in normal cells without endosomal pathology did not affect exosome release or accumulation of endosomes, suggesting that homeostatic exosome release can be maintained in diploid cells even with a perturbation in the expression of a protein involved in exosome generation. CD63 levels may be particularly relevant to the formation of more ILVs when the endosomal-exosomal system is disrupted, as it is in DS.

These findings support our idea that the release of more exosomes by a DS cell allows the cell to regulate its endosomal pathway, albeit not to the point that normal endosomal function can be maintained (Gauthier et al., 2017). Suggesting that this may be a more generalized phenomenon, a recent study reported higher levels of another member of the tetraspanin family, tetraspanin-6, in the brains of AD patients (Guix et al., 2017). In the same study, tetraspanin-6 overexpression was linked *in vitro* to the generation of more exosomes (Guix et al., 2017). We propose that in DS, where endosome dysfunction begins before birth (Cataldo et al., 2000), greater ongoing exosome release moderates, but is not sufficient, to prevent endosomal pathology (Figure 2). These findings in DS emphasize the importance of endosomal-exosomal regulation as a risk factor for neurodegenerative disease and serve to reiterate the interdependence of the endosomal and exosomal pathways.

### Exosome Production Is Compromised by APOE4 Expression

Of the three alleles of apolipoprotein E (APOE) (Mahley, 1988; Mahley and RallJr, 2000), APOE4 is the single most important genetic risk-factor for AD (Corder et al., 1993; Farrer et al., 1997; Bu, 2009; Liu et al., 2013). APOE3 is risk-neutral for AD (Corder et al., 1993; Mahley and RallJr, 2000; Liu et al., 2013) and APOE2 expression is associated with a lower risk of AD (Corder et al., 1994; Liu et al., 2013). Multiple mechanisms appear to drive the pathogenic effects of the APOE4 allele in the brain, including enhancing Aβ deposition while reducing Aβ clearance and degradation; modulating synaptic integrity; modulating cholesterol levels in the brain and the availability of cholesterol and other lipids to neurons; and inducing changes in reactive O_2_ scavenging in the CNS (Strittmatter et al., 1993; Ma et al., 1994; Bales et al., 1997; Higgins et al., 1997; Haan et al., 1999; Holtzman et al., 2000; Shibata et al., 2000; Bu, 2009; Kim et al., 2009; Kolovou et al., 2009; Andrews-Zwilling et al., 2010; Verghese et al., 2011; Villemagne et al., 2011; Leung et al., 2012; Mahley and Huang, 2012; Andrews et al., 2013; Liu et al., 2013; Reinvang et al., 2013; Zlokovic, 2013; Rodriguez et al., 2014). In the absence of a dementia diagnosis, human APOE4-carriers still display structural and functional differences within regions of the hippocampus and cortex, and cognitive decline compared to age-matched non-carriers (Bookheimer and Burggren, 2009; Filippini et al., 2009; Olofsson et al., 2010; Sheline et al., 2010; Wisdom et al., 2011; Liu et al., 2013; Di Battista et al., 2016), and mouse models show cognitive deficits linked to APOE4 expression without AD pathology (Leung et al., 2012; Peng et al., 2017; East et al., 2018).

**FIGURE 3:**
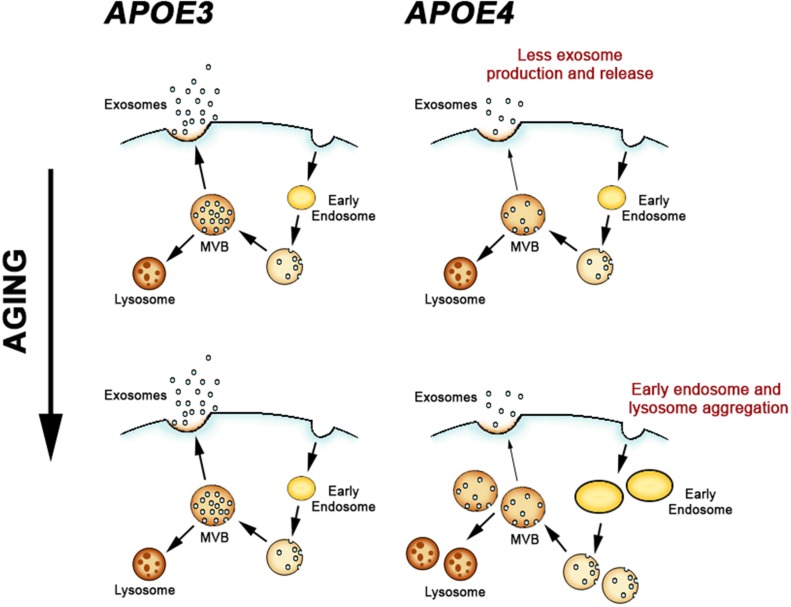
Apolipoprotein E-genotype effects on the endosomal and exosomal pathways. APOE4 expression leads to a deficiency in exosome generation and release in the brain, which subsequently leads to pathogenic alterations in early endosomes and lysosomes during aging.

Mice humanized for the expression of APOE4 do not develop β-amyloid or tau pathology (Wang et al., 2005). However, expression of APOE4 is sufficient to drive dysfunction of the neuronal endosomal–lysosomal pathway in these mouse models in comparison to APOE3 (Nuriel et al., 2017; Zhao et al., 2017). Cataldo et al. (2000) first suggested that APOE4 expression might impact neuronal endosomes when they reported more robust early endosome changes in cortical neurons when comparing APOE4 to APOE3 early stage AD patients. In a number of aspects the APOE4-driven endosomal disruption resembles that seen in DS, including an increase in size and a proliferation in the number of early endosomes (Nuriel et al., 2017; Figure 3). However, the DS endosomal pathway alterations appear to be independent of age and the overexpression of APP that is essential to this phenotype is sufficient to drive endosomal pathway changes in multiple cell types, both *in vivo* and *in vitro* (Cataldo et al., 2003; Salehi et al., 2006; Jiang et al., 2010). This appears not to be the case with APOE4 expression, where the *in vivo* neuronal early endosomal phenotype requires aging. Comparing layer II/III pyramidal neurons of the cingulate cortex of APOE4 to APOE3 mice, early endosomal changes were apparent at 18 months of age but not at 12 (Nuriel et al., 2017). The timing and aging-dependence of early endosome changes in APOE4 mice is particularly intriguing given our subsequent study examining APOE4 effects on brain exosome levels and production (Peng et al., 2019). In aged, non-AD human brain and brain tissue from humanized APOE mice, APOE4 expression decreased brain exosome levels (Peng et al., 2019). The mouse findings, however, showed that this decrease in brain exosome levels is aging-dependent, apparent at 12 but not evident at 6 months of age. 12 months of age is earlier than when endosomal changes were seen (Nuriel et al., 2017). Thus, a reduction in brain exosome levels precedes the neuronal endosomal changes in the brain of APOE4 expressing mice (Figure 3).

In contrast to the findings from DS mouse models, APOE4 expression results in a downregulation of exosome generation and secretion from the endosomal pathway. As we showed in Peng et al. (2019), APOE4 expression reduced both TSG101 and rab35 expression in the brains of aged mice. Decreasing the expression of ESCRT proteins such as TSG101 reduces exosome secretion *in vitro* (Colombo et al., 2013), and inhibition of rab35 *in vitro* leads to impaired exosome secretion by inhibiting fusion of the MVB with the cell membrane (Hsu et al., 2010). An interpretation of these findings from APOE4 mice that is consistent with these prior studies is that exosome biogenesis and secretion are reduced in the brain. Based upon the data from the mouse models, APOE4 expression appears to first disrupt exosome production, followed by broader neuronal endosomal–lysosomal system alterations.

The mechanism connecting expression of the lipid-carrier APOE4 with reduced expression of TSG101 and rab35, and therefore reduced exosome production, is not known. However, because APOE4 compared with APOE3 expression disrupts neuronal and peripheral cholesterol metabolism (Sing and Davignon, 1985; Michikawa et al., 2000), and membrane-lipid changes may compromise the endosomal-exosomal pathway, we examined EV lipids from the brains of humanized APOE mice. We found higher cholesterol levels in the EVs isolated from the brain of APOE4 compared with APOE3 mice, differences that likely reflect altered lipid metabolism within the endosomal pathway from which exosomes originate (Peng et al., 2019). The accumulation of lipids, including cholesterol, in endosomal and lysosomal vesicles occurs in AD and is pathogenic in a subset of genetic lysosomal storage disorders (Distl et al., 2001; Kuech et al., 2016). Increased membrane cholesterol levels, which can result in the secondary accumulation of sphingolipids, can disrupt the endosomal pathway affecting both the morphology and motility of endosomal compartments (Lebrand et al., 2002; Marquer et al., 2014). The functions of several rubs are known to be modulated by endosomal cholesterol levels (Choudhury et al., 2004; Glodowski et al., 2007). For example, endosomal cholesterol accumulation can disrupt MVB mobility and fusion by altering the function of rab7 (Lebrand et al., 2002; Chen and Lamb, 2008). Thus, APOE4-driven changes in cholesterol metabolism and therefore intracellular cholesterol levels may be directly associated with compromised exosome production (Peng et al., 2019). While it has not been determined whether rab35’s function is also affected by cholesterol levels, the changes in rab35 levels in the brain of APOE4 mice (Peng et al., 2019) warrant further studies to determine whether it too is regulated by cholesterol levels in the MVB.

Showing that APOE4 leads to a failure in brain exosome production in an *in vivo* model lacking hallmark AD pathology (Peng et al., 2019) adds further to the evidence that the endosomal–lysosomal system within neurons is extensively disrupted by the expression of this allele (Nuriel et al., 2017). Functional changes within the endosomal–lysosomal system can result in the accumulation of debris in neuronal endosomes and lysosomes (Nixon, 2004), and it appears that dysfunction of this system is an important factor contributing to neuron vulnerability in multiple neurodegenerative disorders, including AD (Nixon, 2005; Schreij et al., 2016). We have proposed that releasing endosomal material into the extracellular space via exosomes can be an important mechanism by which neurons remove endosomal–lysosomal pathway debris (Peng et al., 2019). Indeed, in DS a failure in exosome production worsens the endosomal pathway disturbances (Gauthier et al., 2017). APOE4-driven failure of the neuronal endosomal–lysosomal and exosomal pathways during aging is likely to disturb essential functions of this system, including the efficient degradation of unneeded cellular materials, thus contributing to the risk of neurodegenerative diseases such as AD (Nixon, 2017).

## Conclusion

We have argued that in two AD relevant systems, DS and APOE4 expression, there is strong evidence linking alterations in the endosomal pathway with changes in exosome biology. This interrelationship has been demonstrated in neuronal systems, including the intact brain. These findings are consistent with a model in which flux through the exosomal pathway – i.e., ILV generation and fusion of the MVB with the cell surface to release exosomes – is an essential component of endosomal pathway integrity. In the case of DS, exosome biogenesis appears to be increased to partially compensate for increased endosomal pathway activity. APOE4 expression appears to reduce exosome production with a subsequent impact on the endosomal–lysosomal pathway. While it cannot be ruled out that in the diseased brain the protective effects of the exosome secretion on the endosomal pathway may be offset by the spread and seeding of toxic materials associated with exosomes, we would argue that it is misnomer to discuss exosomes exclusively in the context of promoting the spread and propagation of pathology in neurodegenerative disorders (Howitt and Hill, 2016; Xiao et al., 2017). Indeed, while reducing exosome secretion has been suggested as a potential therapeutic intervention for AD (Dinkins et al., 2014; Asai et al., 2015), our experimental findings in DS models (Gauthier et al., 2017) would argue that such an intervention would lead to greater endosomal pathway pathology, which is likely to be detrimental. Detrimental consequences of limiting exosome production have been shown in an *in vitro* model of α-synuclein pathology (Fussi et al., 2018) and *in vivo* in a transgenic mouse overexpressing a mutant form of the toxic protein TDP-43 (Iguchi et al., 2016). Finally, we have interpreted our findings with APOE4 expression to argue that enhanced exosome production would be protective, which emphasizes the risk of a therapeutic paradigm involving reduced exosome secretion. In summary, exosome production may be protective through the removal of endosomal–lysosomal material from a vulnerable neuron, where this material can be removed from the brain or degraded by support cells. Additionally, exosome secretion can help maintain the homeostatic function of the endosomal–lysosomal and autophagic pathways by offering an additional pathway for the flux of material when lysosomal degradation is insufficient. These beneficial effects of exosome production are most apparent to the neuron secreting the exosomes and are likely to be important in mitigating aging vulnerability to a neuron’s endosomal-autophagy-lysosomal system and the vulnerability this systems sees due to AD.

In this review, we argue that the interrelationship between the endosomal-autophagic-lysosomal system and exosome release has important implications for the survival of neuronal cells. The potential for exosomes to relieve neurons of accumulated, toxic material argues for the importance of developing drugs that can enhance their release. While such compounds are not currently available, greater insight into the biology that drives exosome production as well as exosome clearance in the brain will hopefully guide the therapeutic strategies that can maintain or, in a neurodegenerative disease, restore the integrity of the endosomal-exosomal pathway. Solid evidence has emerged that maintaining the integrity of the overall lysosomal system is critical for the wellbeing of neurons. Tapping into the exosomal pathway appears to be one way to support the stability of this system during aging and with disease.

## Author Contributions

PM and EL conceived the review and wrote the manuscript.

## Conflict of Interest

The authors declare that the research was conducted in the absence of any commercial or financial relationships that could be construed as a potential conflict of interest.
